# Adsorption characteristics of metronidazole on CoZr-LDH and its GO nanocomposite: Experimental and theoretical study

**DOI:** 10.1016/j.heliyon.2025.e42396

**Published:** 2025-02-01

**Authors:** Edris Jamshidi, Fateme Fathabadi, Faranak Manteghi, Rahime Eshaghimalekshah

**Affiliations:** a Research Laboratory of Inorganic Chemistry and Environment, Department of Chemistry, Iran University of Science and Technology, 16846-13114, Tehran, Iran; b Department of Chemistry, Faculty of Chemistry, University of Semnan, Semnan, Iran

**Keywords:** LDH, Nanocomposite, Metronidazole, Adsorption, Graphene nano materials (GNM)

## Abstract

CoZr-LDH and its Graphene Oxide (GO) nanocomposite were synthesized by mechanochemical and hydrothermal methods, respectively, and were applied to absorb metronidazole as a pollutant antibiotic from an aqueous solution. In this study, Tetravalent and divalent metal cations were employed. The LDH and LDH/GO were characterized by SEM, EDS, IR, BET, and XRD analyses. In the adsorption process of metronidazole using both the CoZr-LDH and CoZr-LDH/GO nanocomposite as adsorbents, parameters such as initial solution pH, adsorbent dose, initial drug concentration, and contact time were optimized to obtain the maximum adsorption capacity. The adsorption performance of CoZr-LDH and its nanocomposite with graphene oxide (GO) was thoroughly investigated for the removal of metronidazole (MNZ), a significant environmental contaminant. CoZr-LDH demonstrated optimal adsorption, yielding an experimental qe value of 189.1376 mg g-1. Conversely, the CoZr-LDH/GO nanocomposite exhibited superior adsorption performance under optimum conditions, achieving a remarkable experimental qe value of 906.5688 mg g-1.

The adsorption isotherms, including Langmuir, Freundlich, and Redlich-Peterson models, were fitted to the experimental data to describe the interaction mechanisms and surface characteristics. Additionally, kinetic studies involving pseudo-first-order and pseudo-second-order models were performed, revealing critical insights into the adsorption process. Post-adsorption characterization confirmed the successful interaction between MNZ and the adsorbents. This study emphasizes the potential of GO-enhanced CoZr-LDH for effective removal of metronidazole, addressing its status as a critical pollutant in wastewater.

To comprehensively study the adsorption process in CoZr-LDH and CoZr-LDH/GO, theoretical analyses were conducted using Monte Carlo computational method. This approach, known for simulating numerous particle trajectories and statistical averaging, allowed for an in-depth examination of adsorption mechanisms under various conditions. The findings, validated against experimental data, enhanced model accuracy and provided deeper insights into adsorption behaviors in complex environments.

## Highlights

• **First-time synthesis of CoZr-LDH and its composite with graphene oxide (GO)** using a green, solventless mechanochemical method.
• **Novel Study of metronidazole adsorption** on CoZr-LDH/GO reveals key interactions of this environmental antibiotic pollutant.
• **First-time evaluation of adsorption capacity,** highlighting structural benefits and pollutant interaction mechanisms.
• **Quantum and Monte Carlo simulations** analyze LDH and LDH/GO adsorption, providing key insights into adsorption efficiency.

## Graphical abstract

**Figure:**
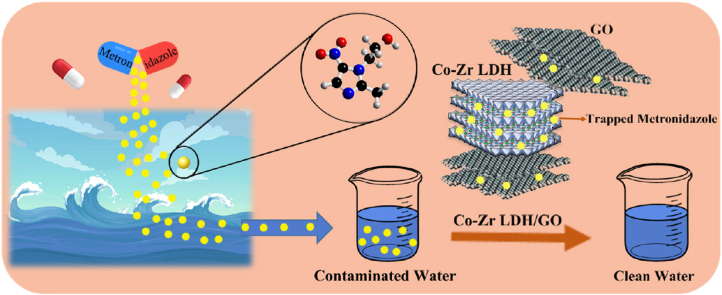

## 1 Introduction

Due to human, agricultural, and industrial activities, significant volumes of hazardous organic and inorganic pollutants have found their way into wastewater systems and aquatic environments across the globe [[1], [2], [3], [4], [5], [6]]. The discovery of unexpected pollutants in sewage and surface water, including heavy metals, drugs, cosmetics, dye, and household goods, have garnered significant interest [[7], [8], [9], [10], [11], [12], [13]]. Most of the medicinal compounds found in water include antibiotics, painkillers, antidepressants, antihistamines, anti-inflammatory medications, anticancer medications, etc [1,14]. Antibiotics are present in surface water, sewage, groundwater, and drinking water in amounts ranging from a few ng/L to g/L. In addition, the use of antibiotics in medicine and veterinary medicine has led to their accumulation in aqueous environments [15]; however, the main source of these antibiotics released is drug factories [16].

Given the growing concerns of the environment and the general public, the development of efficient technology for the synthesis of antibiotics is desperately needed [[17], [18], [19]]. Moreover, both people and animals have poor metabolization and absorption of antibiotics. This long-term function leads to the development and spread of antibiotic-resistant bacteria and antibiotic-resistance genes that have severe effects on the environment [20].

Because the structures of these compounds, whether from older or newer generations, contain benzene rings, they are hazardous to aqueous solutions; therefore, conventional purification methods typically fail to remove them, and as a result, they become increasingly hazardous and unusable through interactions with chemicals employed in water refinement [20]. As an antibiotic and food additive, metronidazole is used to treat anaerobic and protozoan microorganisms. Effluents from the meat industry, drinking water, and wastewater treatment plants have all frequently contained it. Its limited biodegradability and high water solubility give rise to serious hazardous consequences [1].

Studies have indicated that metronidazole can be found in surface waters and wastewater, often as a result of human and veterinary pharmaceutical waste-aquatic environments raises serious ecological concerns as it can disrupt microbial communities essential for biogeochemical processes [21,22]. Additionally, male's potential genotoxic and mutagenic effects have been documented, raising concerns about chronic exposure in both aquatic organisms and humans [23,24]. Such health risks are necessary into effective removal strategies to mitigate its environmental persistence and ensure ecosystem safety [25,26]. Wastewater can be treated using a variety of techniques, including oxidation, electrical breakdown, ozonation, biodegradation, and photocatalytic decomposition [27]. However, adsorption is the most suitable technique because of its restricted transfer to aquatic systems and inhibiting harmful effects [[28], [29], [30]]. Furthermore, Utilizing gamma radiation, for Metronidazole decomposition and mineralization, the byproduct toxicity of which was greater according to previous results [18]. Adsorption is a water treatment procedure that removes a wide spectrum of organic and inorganic pollutants. It is widely utilized in both laboratories and the field. The use of this technique in urban water treatment is widely established [31].

Layered double hydroxides of two-dimensional anionic clays consist of brucite-like layers and exchangeable charge-balanced interlayer anions with the structural formula [M^2+^_1-x_M^3+^_x_(OH)_2_]^x+^(A^n−^)_x/n_·mH_2_O, where M^2+^ and M^3+^ are divalent and trivalent cations, respectively, A is an interlayer anion with charge n, and x denotes the molar fraction of trivalent cations [[32], [33], [34]]. The structure of interlayer spaces is likewise stabilized by water molecules [35,36]. The uses of LDHs in various fields such as catalysis, flame retardant, adsorption, drug delivery, supercapacitors, fuel cells, electrochemical sensors, and biosensors have been reported, however, most of their applications are as adsorbents [35,37,38].

LDH materials are suitable for the effective adsorption of anionic pollutants due to their large surface area, flexibility, and strong anion exchange capacity, in the interlayer gaps [31]. LDHs have the flexibility to adapt their structure and are easily recycled and reused [35]. LDHs have three mechanisms to adsorb anions in an aqueous solution: anion exchange, direct adsorption, and intercalation of the LDH [35]. Due to the highly adjustable structure of LDHs, their exchangeable anions can be easily replaced with organic anions in addition to inorganic anions [39], However, monovalent anions are better than any other type of anion, whether organic or inorganic, because they are simple to replace [32]. The ion exchange capacity of LDHs depends on the nature of the initially mediated anions and the charge density of the layer (ratio of M^2+^/M^3+^([35].

**Fig. 1:**
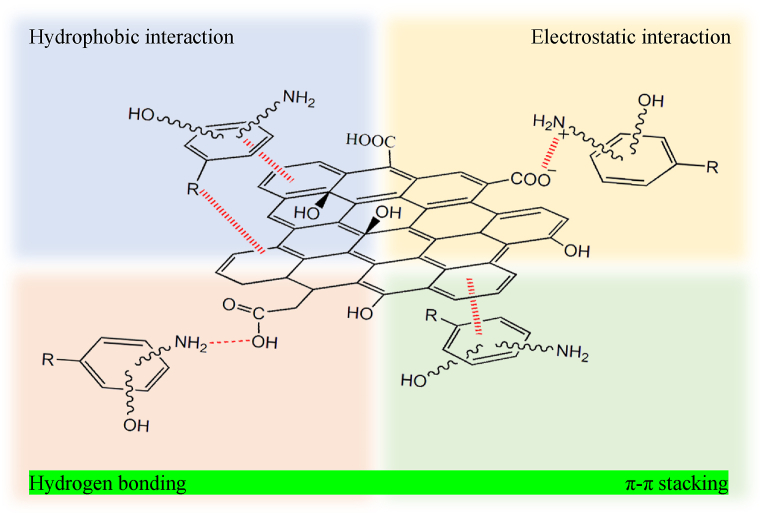
An overview of various interactions between antibiotics on GNMs.

Graphene nanoparticles are highly soluble in water, and the dispersion of graphene nanoparticles in aqueous media causes secondary contamination and makes it difficult to separate these materials after modification. Graphene nanosheets are composites of different materials that reduce bioavailability and increase adsorption efficiency [40]. Antibiotic molecules with aromatic rings can have 4 types of interactions with graphene (Fig. 1).

1. π-π stacking of antibiotic molecules with aromatic graphene ring π electrons.
2. Hydrogen bonding of antibiotic molecules with hydrogen ions from carboxyl or hydroxyl groups of GO.
3. Hydrophobic interactions of antibiotic molecules with hydrophobic groups on GNMs.
4. Electrostatic interactions of antibiotic molecules with carboxyl groups at different pH values include cation attraction (pH < pKa of antibiotic molecules) or anion attraction (pH > pKa of antibiotic molecules) [40].

In this study, we combined CoZr-LDH, which was synthesized in a different way than that in previous reports, with graphene. This adsorbent was used two adsorbents to remove metronidazole from the aqueous solution and then compared the two adsorbents in terms of adsorption capacity. The effects of different optimal conditions were also examined. According to the findings, the adsorption capability of the CoZr-LDH/GO nanocomposite over CoZr-LDH increased due to the increase of functional groups and trapping of metronidazole between graphene layers and LDH.

## 2 Materials and methods

### 2.1 Chemicals

The cobalt and zirconium salts, NaOH, H_2_SO_4_(98 %), HCl, H_2_O_2_, methanol, urea, NaNO_3_, KMnO_4_ and graphite were purchased from Merck. Metronidazole (99 %) was purchased by the Darupakhsh, Iran company. Every step of the project involved the usage of deionized water.

### 2.2 Synthesis of CoZr-LDH

A mechanochemical technique was used to synthesize Co-Zr LDH. To put it briefly 2.1 g of ZrCl_4_ and 6.12 g of Co(NO_3_)_2_.6H_2_O (zirconium and cobalt salts) were added to an agate mortar. After 15 min, 2.76 g of NaOH was gradually added and

Ground for 50 min. Here, it turned from a pale pink to a brown hue. The resulted dark precipitate was washed 3 times with deionized water and dried in a vacuum oven after complete removal of the disturbing ions.

### 2.3 Synthesis of GO

The Hummers' method was used to synthesize graphene, as reported in the article [41].

### 2.4 Synthesis of CoZr-LDH/GO nanocomposite

A hydrothermal method was used for the synthesis of the CoZr-LDH/GO nanocomposite. Initially, 0.3 g of graphene was dissolved in 80 mL of methanol and dispersed by sonication for 1 h. Fifteen millimoles of zirconium salt with 34 mmol of cobalt salt were ground well in an agate mortar, added to a graphene solution with 0.7 g of urea, and dispersed by an ultrasonic probe for 1 h. The black solution, after being moved to a Teflon autoclave, was heated at 210 °C for 12 h. After washing with methanol and filtration, the precipitate was dried in an oven and then placed in a vacuum oven overnight [42,43].

### 2.5 Adsorption study

To evaluate and compare the CoZr-LDH and CoZr-LDH/GO nanocomposite adsorbents with the best efficiency for metronidazole adsorption, at a concentration of 2000 ppm, the first metronidazole stock solution was prepared. Then, the required concentrations of metronidazole were prepared by continuously retiring the standard solution with deionized water. Using 0.1 M, HCl, and NaOH solutions to adjust the pH in the range of 4–10, the impact of different combinations on metronidazole absorption was examined. To evaluate the adsorbent dose, the portions of 0.001, 0.002, 0.005, 0.01, and 0.03 g of adsorbent were added to the metronidazole solution (at the optimal concentration and pH), after which the effect of the adsorbent was investigated. The optimal metronidazole solution was sampled at 35–60 min, after which the effect of time on the adsorbents was examined. In all the optimization steps after shaking at 250 rpm, the adsorbents were separated via centrifugation and the adsorption range was recorded via a UV–VIS spectrophotometer in the range of 200–500 nm.

Equation (1) was used to calculate the adsorbents' capacity for adsorption.

(1) $$q_{e}=\left(C_{o}‐C_{e}\right)V/m$$

where C_o_ and C_e_ are the initial and equilibrium concentrations of MNZ (mg/L), respectively, V is the volume of the used solution (L), and m is the mass of the used adsorbent (g).

#### 2.5.1 Kinetic study

To approach more comprehensive data on the adsorption kinetics for further interpretation of the process, kinetic approaches, such as pseudo-first- and pseudo-second-order models, were examined using the MNZ adsorption data and equations (2), (3).

(2) $$\text{Pseudo}‐\text{first}\;\text{order}\;\;\text{model}\;\ln\left(q_{e1}‐q_{t}\right)=‐k_{1}t+{\text{lnq}}_{e1}$$

(3) $$\text{Pseudo}‐\text{second}\;\text{order}\;\;\text{model}\;t/q_{t}=1/k_{2}q_{e2}^{2}+t/q_{e2}$$

Where q_e1_ and q_e2_ are the amounts of MNZ adsorbed (mg g^−1^) at equilibrium in pseudo-first and pseudo-second-order models, and the q_t_ is the amount of adsorbed MNZ equilibrium in the specific time, The constants for pseudo-first and pseudo-second order reactions, respectively, are k_1_ (min^˗1^) and k_2_ (g mg min^˗1^).

#### 2.5.2 Isoterms

Three adsorption isotherms, Langmuir and Freundlich and Redlich-peterson were employed to describe the interaction of adsorbent species with adsorbents. The following assumptions are based on the Langmuir isotherm.

1. Adsorption takes place on the adsorbent outer surface at particular homogenous spots.
2. All places are equal
3. There is no interaction between adsorbent molecules at adjacent locations
4. Adsorption is one layer and no other adsorption occurs after that [44].

The Langmuir isotherm is predicated on the theory that adsorption takes place on the adsorbent's outer surface through a number of unique homogenous sites, all the sites are equivalent, only monolayer adsorption takes place, and adsorbate molecules have no interaction on adjacent sites. According to the Freundlich adsorption isotherm, adsorption is assumed to occur as a multilayer with interactions between adsorbed molecules. Adsorption occurs on heterogeneous surfaces and reversibly [45].

The mathematical relationships of the Langmuir and Freundlich adsorption isotherms are given in equations (4), (5), (6) respectively.

(4) $$C_{e}/q_{e}=1/q_{m}k1+C_{e}/q_{m}$$

(5) $${\text{lnq}}_{e}=1/n\;{\text{lnC}}_{e}+\ln\;k_{f}$$

(6) $$q_{e}=K_{R‐P}\;C_{e}/1+\alpha_{R‐P}\;C_{e}^{\beta }$$

k_f_ and k_l_ are the kinetic constants for the Freundlich model and the Langmuir model, respectively. In equation 6, K _R–P_ is Redlich–Peterson constant (L g ^−1^), α_R–P_ is constant (L mg ^−1^)^−β^, β is exponent that lies between 0 and 1.

The Redlich–Peterson isotherm is widely recognized for its ability to bridge the gap between the Langmuir and Freundlich models, making it suitable for both homogeneous and heterogeneous systems. Its versatility allows it to describe mixed adsorption mechanisms, indicating that the process does not adhere strictly to ideal monolayer adsorption [46].

Typically, the value of β ranges between 0 and 1; however, when β is equal to or greater than 1, the adsorption behavior can be more accurately described by the Langmuir isotherm model.

### 2.6 Computational calculations

#### 2.6.1 Quantum method

First, to obtain optimized geometric structures, the structures of NO_3_^−^, CO_3_^2−^, H_2_O, GO, metronidazole (MNZ), CoZr, and CoZr-LDH (NO_3_^−^, CO_3_^2−^, and H_2_O) were designed and optimized by using the DMol^3^ module via DFT-D correction [47,48]. Also, Task: geometry optimization employing the generalized gradient approximation of the Perdew-Burke-Ernzerhof (GGA-PBE) functional and DND (similar to 6-311G∗) along with Basis set; Medium was used in Materials Studio 2017 software [49,50]. In this study, Grimm, DFT-D, Spin unrestricted and Core treatment; Effective core potentials were applied, and Multipolar expansion; Quadrupole with 50 SCF cycles and Global orbital cutoff were used in this software [51]. The functional groups including hydroxyl and carboxyl groups, were randomly bonded to the carbon atoms of GO.

#### 2.6.2 Monte Carlo adsorption locator method

To create CoZr-LDHs, 4 molecules NO_3_^−^, CO_3_^2−^ and H_2_O interacted between CoZr layers. Then, a Monte Carlo adsorption locator with Forcefield universal was utilized to adsorb 3 molecules of GO on CoZr-LDH via Materials Studio 2017 software. Finally, to adsorb MNZ on CoZr-LDH and CoZr LDH/GO, a Monte Carlo adsorption locator (forcefield) is accustomed to adsorb 3 molecules of GO [52].

### 2.7 Characterization methods

#### 2.7.1 Instruments

A Philips X'pert device was used to obtain powder X-ray diffraction (PXRD) patterns, with monochromatic Cu-kα radiation (λ = 1.54056 °A), and X-pert software was utilized for generating simulated XRD powder patterns based on reference cards. FT-IR data were recorded using an AVATAR device from Thermo Scientific, utilizing KBr disc technique and covering the range of 400–4000 cm^−1^. The UV_VIS spectrum was measured at room temperature using a SHIMADZU spectrophotometer. A PARTICLE METRIX device measured zeta potential data at different pH values. The morphology of the adsorbents (SEM) and their elemental distribution (EDS) were recorded by a MIRA3 scanning electron microscope from TESCAN Company, in the power of 0–50 keV with a distance of 19 mm, it's worth mentioning that the take-off angle was 35°. Specific surface measurements (BET) of the CoZr-LDH and CoZr-LDH/GO nanocomposite at their degassing temperature were performed by a BELSORP MINI device from the MICROTRAC BELCROP Company.

#### 2.7.2 Characterization results

**Fig. 2:**
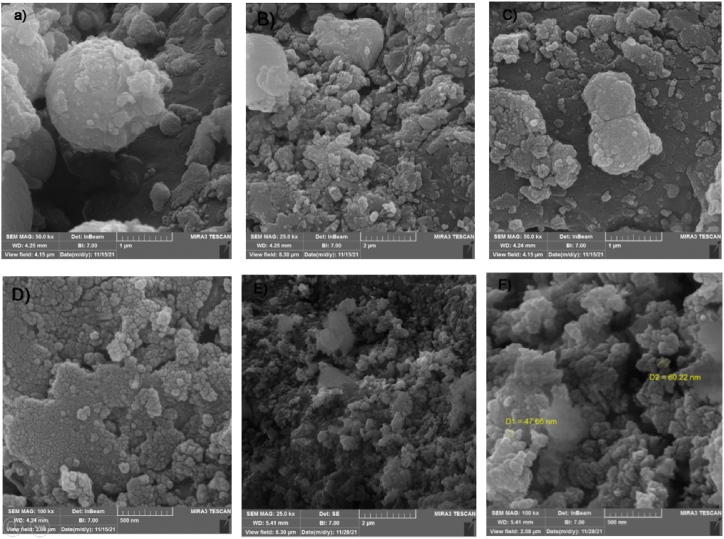
A–D) SEM images of Co-Zr LDH. E, F) SEM images of Co-Zr LDH/GO.

Scanning electron microscopy (SEM) was used to analyze the adsorbents' surface morphology at different magnifications and is shown in Fig. 2. As illustrated in Fig. 2E and F, the LDH layer-by-layer structure and GO sheets settled between the layers. As a result, the semitransparent paper-like structures are GO sheets.

**Fig. 3:**
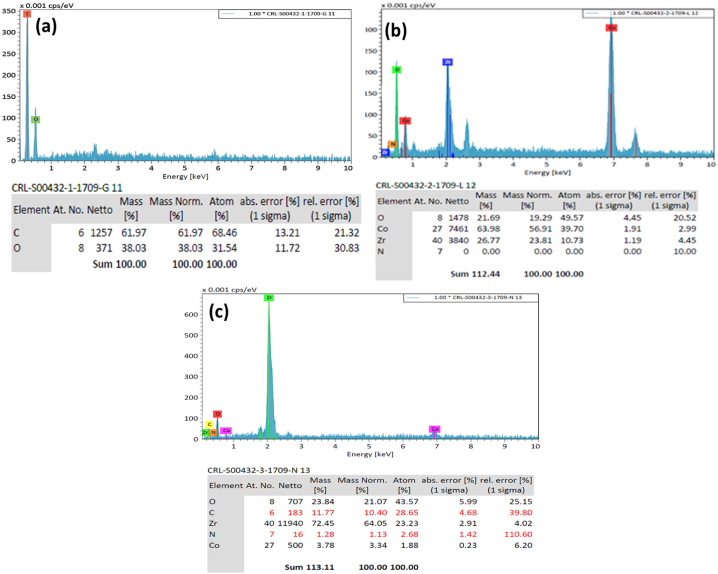
EDS diagrams of a) GO b) Co-Zr LDH and c) Co-Zr LDH/GO nanocomposite.

The elemental distributions of the CoZr-LDH, CoZr-LDH/GO nanocomposite, and graphene oxide structures recorded by EDS analysis are shown in Fig. 3. EDS analysis based on our expectations showed that CoZr-LDH is composed of Co, Zr, O, and N; that graphene oxide is composed of C and O; and that the CoZr-LDH/GO nanocomposite is composed of O, C, Zr, Co and N.

**Fig. 4:**
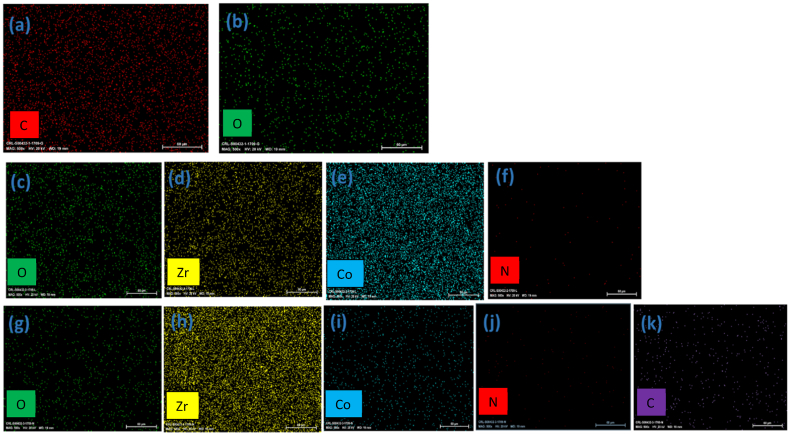
Elemental mapping of a,b) Go, c-f) Co-Zr LDH and, g-k) Co-Zr LDH/GO.

Energy-dispersive X-ray spectroscopy (EDS) mapping of the samples shown in Fig. 4. For graphene oxide (GO), which primarily contains carbon (C) and oxygen (O), EDS mapping reveals a homogeneous distribution of these elements. The oxygen content typically reflects the oxygenated functional groups on the surface of graphene oxide, such as hydroxyl, carboxyl, and epoxy groups, which are key to its chemical reactivity and interaction with other materials. The uniformity of the carbon and oxygen distribution also highlights the structural integrity of GO sheets.

In the case of Co-Zr LDH and the Co-Zr LDH/GO nanocomposite, EDS mapping provides detailed insights into the distribution of cobalt (Co), zirconium (Zr), carbon (C), oxygen (O), and, in the nanocomposite, nitrogen (N). For Co-Zr LDH, the mapping confirms the even dispersion of Co and Zr within the layered hydroxide matrix, while carbon and oxygen are also present due to the organic and hydroxyl components. In the Co-Zr LDH/GO nanocomposite, EDS shows the successful integration of GO, with nitrogen being introduced as a result of functionalization. The mapping reveals a well-dispersed distribution of all elements, confirming the successful formation of a uniform nanocomposite material with enhanced properties.

**Fig. 5:**
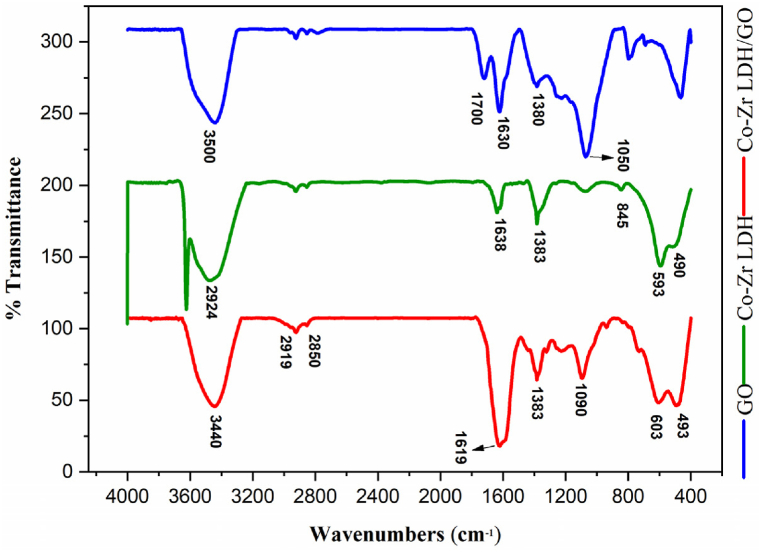
IR spectra of graphene oxide (blue top spectrum), Co-Zr LDH (green middle spectrum), and the Co-Zr LDH/GO nanocomposite (red bottom spectrum).

As shown in Fig. 5, in the blue top spectrum, the peaks at 1380 and 1050 cm^−1^ correspond to the tensile and flexural vibrations of the C−O, respectively. The peak at 1700 cm^−1^ belongs to the carbonyl group and the peak at 1500–2000 cm^−1^ corresponds to C=C vibrations [53,54]. The flexural vibration of the OH at 3500 cm^−1^ is clearly marked [55]. According to the marked peaks, the formation of GO is clearly understood.

The IR technique was used to determine the characteristics and alignment of the interlayer anion. As shown in Fig. 5, in the green middle spectrum, Peaks at 1383 cm^−1^ and 843 cm^−1^ corresponded to the interlayer nitrate anions' tensile and flexural vibrations. The presence of M-O-M linkages was confirmed by 593 cm^−1^ and 490 cm^−1^ peaks. One band, 1638 cm^−1^, was associated with water's flexural vibration. The water's tensile OH groups were found to be present at 2924 cm^−1^. According to the pointed peaks, the formation of CoZr-LDH were confirmed.

According to Fig. 5, the red bottom spectrum indicates the 2850 and 2919 cm^−1^ peaks related to the C−H bonds of graphene. The 1383 cm^−1^ peak showed carbonate vibrations. The bands at 603 and 493 cm^−1^ affirmed the presence of M−O, and the 1618 cm^−1^ peak was associated with C=N. The formation of the CoZr-LDH/GO nanocomposite was confirmed according to the specific peaks.

**Fig. 6:**
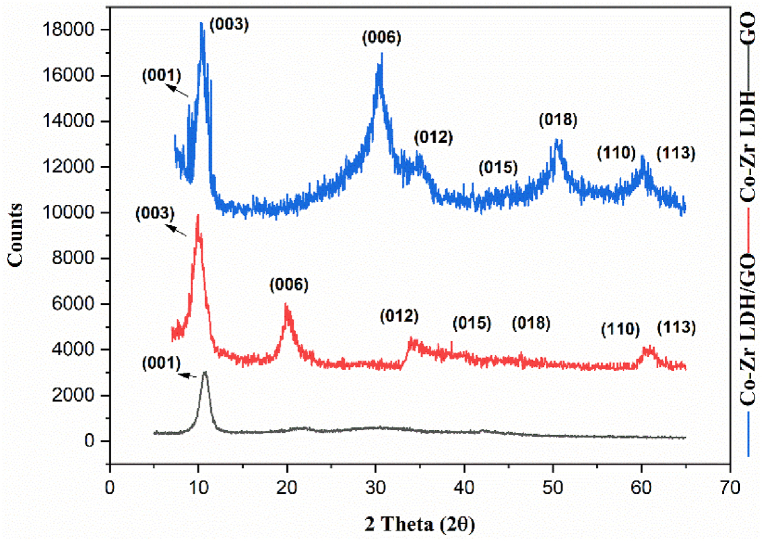
XRD patterns of GO (black, bottom), Co-Zr LDH (red, middle), and Co-Zr LDH/GO nanocomposite (blue, top).

X-ray diffraction (XRD) was performed for the CoZr-LDH, GO, and CoZr-LDH/GO nanocomposite, and the results shown in Fig. 6 were consistent with a previously reported structure [45]. For precise structural analysis, a Philips X'Pert X-ray diffraction (XRD) instrument equipped with Cu-Kα radiation (λ = 1.54 Å) was utilized. The analysis was conducted under operating conditions of 40 kV and 30 mA, with a step size of 0.04° per second for XRD analysis.

The observed XRD pattern displays the distinctive diffraction peaks of the LDH structure by using a hexagonal phase. X-ray powder diffraction (XRD) revealed Co-Zr LDH plates at positions (003), (006), (012), (015), (018), (110) and (113) in 2Ɵ of 11.72, 23.559, 34.653, 39.273, 46.824, 60.229, and 61.619, respectively (Fig. 6), which line up with the characteristic models of the hydrotalcite structure. Moreover, the diffraction peak at (001) (2θ∼10°) corresponds to the GO, and the shift of the (006) peak in the LDH/GO compared to the LDH to the right is obviously due to the compression of LDH (006) and (018) planes during the solvothermal synthetic procedure. The results obtained demonstrate the incorporation of GO into the LDHs and validate a successful synthesis of CoZr-LDH/GO [45,49].

**Fig. 7:**
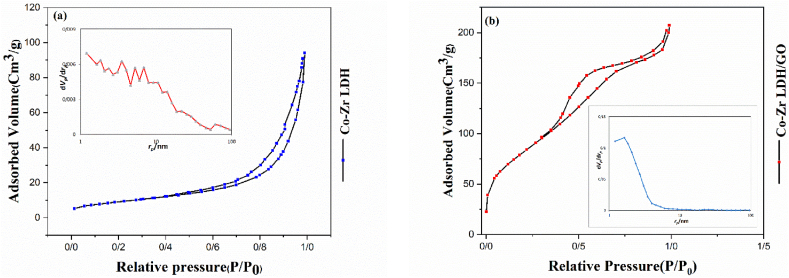
Nitrogen adsorption-desorption isotherms of a) CoZr LDH and b) CoZr LDH/GO. The inset graph shows the pore size distribution curves of the samples.

The porosity data of the LDH and LDH/GO are summarized in Fig. 7. These two hydrotalcite-like samples CoZr LDH and CoZr LDH/GO had surface areas of 32.995 m^2^ g^−1^ and 297.47 m^2^ g^−1^, respectively. The isotherms for the nitrogen adsorption-desorption demonstrated that the addition of GO (inter- or outer layer) increased the surface area.

The physical characteristics, such as the average pore diameter, surface area, and external pore diameter, are summarized in Table 1.

**Table 1:** Surface features of Co-Zr LDH and Co-Zr LDH/GO.

Samples	Surface area (m^2^ g^−1^)	External surface area (m^2^g^−1^)	Average pore diameter (nm)	Total pore Volume (cm^3^ g^−1^)	Adsorptive
Co-Zr LDH	32.995	35.053	17.502	0.1444	N_2_
Co-Zr LDH/GO	297.47	260.96	4.2901	0.319	N_2_

## 3 Results and discussion

### 3.1 Adsorption performance

**Fig. 8:**
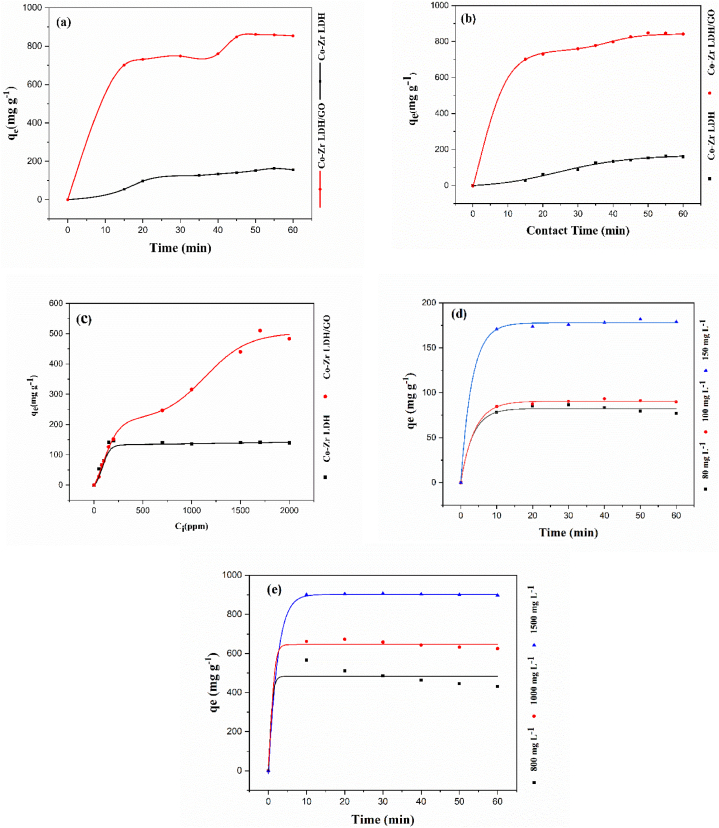
All prepared water-stable samples' adsorption performance a) contact time's impact on adsorption b) initial concentration impact concerning time. c) Result of different initial concentrations on adsorption. d) initial concentration impact concerning time on CoZr LDH. e) initial concentration Effect concerning time on CoZr LDH/GO.

Graphs of the adsorption capacities of CoZr LDH and the CoZr LDH/GO nanocomposite for metronidazole adsorption from aqueous solution are shown in Fig. 8a. The diagrams show that the adsorption capacity of the CoZr LDH/GO nanocomposite is greater than that of CoZr LDH alone. A large difference was observed in the adsorption results and optimum conditions between the two samples in the experimental measurements: CoZr LDH exhibited the best adsorption performance at 150 ppm of MNZ, 0.01 g of adsorbent, pH 6, and with a contact time of 55 min, under these conditions, the experimental q_e_ value was 189.1376 (mg g^−1^). However, these numbers changed in a favorable way when the Co-Zr LDH/GO was used. By using a GO-assisted nanocomposite, the best adsorption performance was observed in 1500 ppm MNZ concentration, 0.001 g of adsorbent powder, at pH 8, and a contact time of 30 min. The experimental q_e_ value was 906.5688 (mg g^−1^) in this study. The outcomes demonstrated that the surface adsorption of the Co-Zr LDH/GO nanocomposite was much greater due to the abundance of oxygen functional groups, which emanated from graphene oxide. Conversely, the π-π interactions between the metronidazole ring and the graphene hexagonal honeycomb lattice increase the adsorption capacity; ultimately, some of the metronidazole molecules are trapped in the nanocomposite structure via space-filling, which is caused by large network area of the graphene lattice.

The time adsorption interaction and initial concentration graphs for these two adsorbents are shown in Fig. 8b. Adsorption took about 55 min for Co-Zr-LDH and about 30 min for CoZr-LDH/GO nanocomposite to reach equilibrium, but to investigate equilibrium fluctuations, adsorption lasted about 60 min. Adsorption began extremely quickly and spontaneously and gradually slowed down until equilibrium was reached. As shown in Fig. 8b, after reaching equilibrium, the adsorption capacity remained almost constant.

#### 3.1.1 Kinetic study

Table 2 displays the findings from the kinetic models.

**Table 2:** Adsorption kinetics parameters for a) CoZr-LDH and b) CoZr-LDH/GO nanocomposite.

Samples (mg g^−1^)	q_e_,exp	Pseudo-first order model				Pseudo-second order model			
		*K*_*1*_*(*min^*−1*^*)*	*q*_*e1*_	*R*^*2*^	***χ 2***	K_2_ (g mg^−1^ min^−1^)	q_e2_ (mg g^−1^)	R^2^	***χ* 2**
CoZr LDH	189.137	6 × 10^−3^	196.6057	0.9151	2.312	1.01 × 10^−4^	263.1579	0.9851	0.351
CoZr LDH/GO	906.57	4.2 × 10^−4^	74.76127	0.9609	1.538	8.07 × 10^−4^	909.0909	0.9974	0. 211

**Fig. 9:**
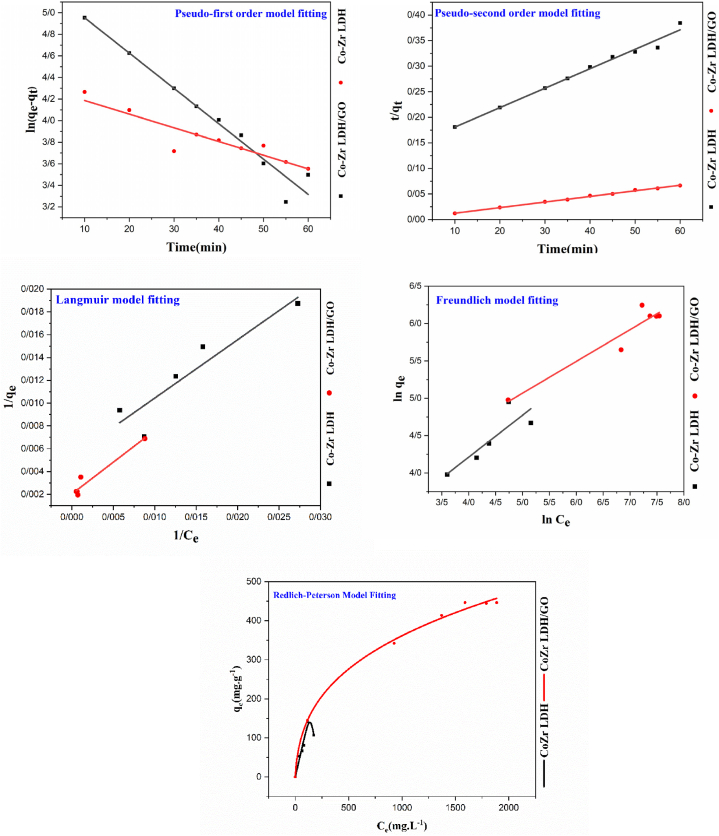
One-dimensional kinetic and isothermal model adjustment.

The kinetic data (q_e1_ and q_e2_) were 196.6057 and 263.1579 for CoZr LDH and 74.76127 and 909.0909 for the CoZr LDH/GO nanocomposite, respectively. As a result, according to the association coefficient (R^2^), the adsorption process for both adsorbents follows the pseudo-second-order kinetic model [56]. The adsorption diagram for both adsorbents is shown in Fig. 9, and also the data that is obtained from the experiments and the pseudo-first order and pseudo-second order kinetic models are reported in Table 2.

For CoZr LDH, the values of k_1_ and k_2_ varied significantly, but the experimental results closely matched the pseudo-second-order kinetics model's values. As such, according to the association factor (R^2^), the pseudo-second-order model describes this process better than the pseudo-first-order model.

In general, while the pseudo-second-order (PSO) model effectively fits the experimental adsorption kinetics data, it provides limited insight into the underlying adsorption mechanisms. Similarly, the Elovich equation appears to capture a range of reaction mechanisms, including bulk and surface diffusion, yet lacks specificity in distinguishing the dominant processes [57,58]. However, in this study, due to the size of the adsorbate molecules and the pore size of the adsorbents, diffusion is unlikely to occur. As a result, it is unnecessary to apply the Elovich equation or other kinetic models, such as the intraparticle diffusion model.

#### 3.1.2 Isotherms

Three adsorption isotherms, Langmuir, Freundlich, and Redlich-Peterson were employed to describe the interaction of adsorbent species with adsorbents. The conformance of the model with experimental data is based on calculated values of (χ^2^) and (R^2^).

Adsorption isotherm data and diagrams for CoZr-LDH and the CoZr-LDH/GO nanocomposite are shown in Table 3 and Fig. 9. All models show good fitting with experimental data, Since the R values were close to each other. The chi-square (χ^2^) is the most effective error value approach for accurately determining isotherm model parameters. Based on the results, the isotherms can be ranked as follows: Redlich-Peterson > Langmuir > Freundlich. Among all the models, the Redlich-Peterson isotherm provides the best fit to the adsorption data, as evidenced by its highest R^2^ values (0.9602 for Co-Zr LDH and 0.997 for Co-Zr LDH/GO) and the lowest χ^2^ values (1.537 for Co-Zr LDH and 0.1362 for Co-Zr LDH/GO) (Table 3). The Langmuir model exhibited lower R^2^ values (<0.96) and higher χ^2^ values (>1.53) compared to the Redlich-Peterson model, indicating a poor fit between the experimental data and the Langmuir model. In contrast, the Redlich-Peterson model, which integrates key aspects of both the Langmuir and Freundlich models, provides a more accurate representation of the adsorption equilibrium.

**Table 3:** Isothermal parameters for a) CoZr-LDH and b) CoZr-LDH/GO nanocomposite.

Samples	Freundlich Model				Langmuir Model				Redlich-Peterson				
	*K*_*F*_*(mg g*^−*1*^*)*	*n*	*R*^*2*^	***χ***^**2**^	q_m_(mg g^−1^)	K_L_ (mg g^−1^)	R^2^	***χ***^**2**^	*K*_*RP*_ (L.g^−1^)	*α*_RP_ (mg.L^−1^) ^*β*RP^	*β*_RP_	*R*^*2*^	***χ***^**2**^
Co-Zr LDH	7.177943	1.785077	0.743	13.47	188.6792	0.010365	0.861	9.74	1.18038	0.00014	0.75	0.960	1.53
Co-Zr LDH/GO	18.9147	2.349624	0.9151	8.23	500	0.003589	0.940	5.77	5.6106	0.1466	0.66	0.997	0.136

The β values (0.75 for Co-Zr LDH and 0.66 for Co-Zr LDH/GO) indicate that metronidazole (MNZ) adsorption on both adsorbents does not follow ideal monolayer adsorption. These findings suggest that multilayer adsorption is the predominant mechanism in the removal of MNZ by Co-Zr LDH and Co-Zr LDH/GO [35].

It stands to reason that enhancing the surface area of these materials would expand the binding sites' volume and, as a result, their ability to bind MNZ.

The monolayer adsorption amounts of Co-Zr LDH and Co-Zr LDH/GO were 188.6792 mg g^−1^ and 500 mg g^−1^, respectively. These outcomes confirm that the adsorption uptake of these two adsorbents was greater than the Langmuir q_e_ value. This means that in addition to monolayer adsorption, multilayer adsorption occurs.

In the Freundlich model, a value of 1/n in Equation (5) shows surface heterogeneity or adsorption intensity, which results in more heterogeneity when approaching zero, and a value less than 1 indicates the desired adsorption [41]. According to the results, the 1/n value is 0.56 for CoZr-LDH and 0.425 for the CoZr-LDH/GO nanocomposite, which indicates a favorable absorption [59].

Surface heterogeneity or adsorption intensity is indicated by the value of 1/n (Eq. (3)). revealing that heterogeneity increases as the value approaches zero. A value of 1/n < 1, indicates favorable adsorption.

#### 3.1.3 pH study

It has been established that an adsorption system may be significantly impacted by the pH of a system. The studies were carried out at various initial pH values in order to ascertain the influence of pH.

Experiments on the pH of metronidazole adsorption from aqueous solution were performed in the range of 4–10 (at low pH values, the adsorption and dissolution of metronidazole has a low yield) [60,61]. Moreover, a dynamic function for hydrogen bonds and electrostatic interactions in the whole adsorption process is proposed [20].

**Fig. 10:**
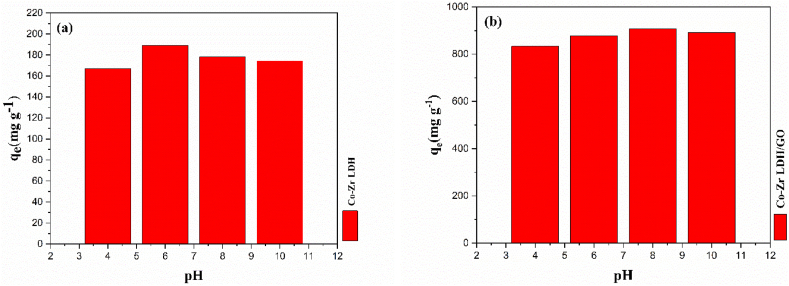
a) pH impact on MNZ removal over CoZr LDH and b) CoZr LDH/GO.

The absorption capacities of CoZr-LDH and the CoZr-LDH/GO nanocomposite increased with increasing pH to approximately 8 and then remained almost constant. As shown in Fig. 10, the maximum adsorption capacities of the CoZr-LDH and CoZr-LDH/GO nanocomposite adsorbents were observed at pH 6 and 8, respectively. At pH values above 8, the binding sites of CoZr-LDH are filled with positive and negative ions, Therefore, the adsorption capacity is reduced [60,62].

**Fig. 11:**
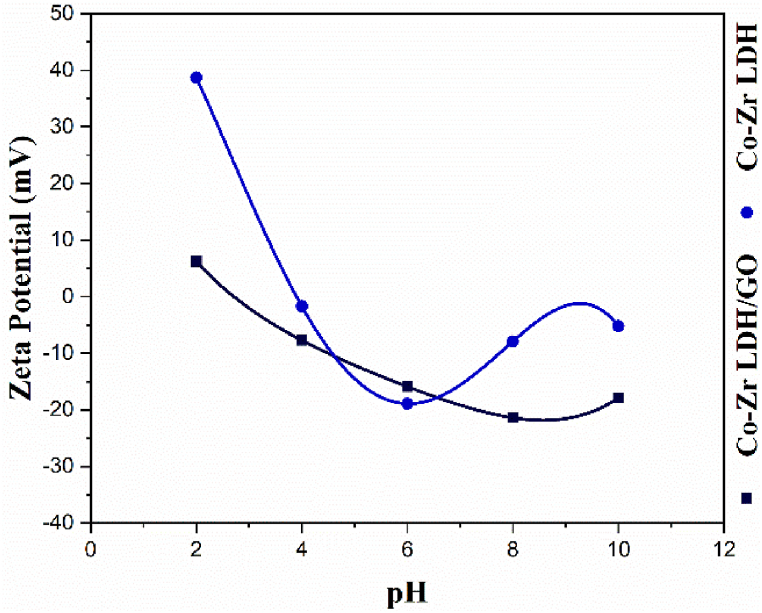
Zeta potential at different pH for a) CoZr-LDH and b) CoZr-LDH/GO nanocomposite.

The pH solution can affect the surface charge of the medium and the equilibrium ionization of the solutes. The pH_zpc_ represents the effect of pH on the surface charge of the medium [45]. As shown in Fig. 11, the pH_zpc_ values of the CoZr-LDH and CoZr-LDH/GO nanocomposite are −21.4 and −18.9, respectively. Due to the inherently positive potential of metronidazole, it is anticipated that adsorption efficiency will be higher when the adsorbent surface carries a negative charge. As depicted in Fig. 11, the surface charge of the adsorbents reaches its most negative potential at pH levels 6 and 8, aligning with the pH values at which the highest adsorption capacities were observed. This correlation suggests that the electrostatic interactions between the positively charged metronidazole molecules and the negatively charged adsorbent surface are maximized under these conditions, leading to enhanced adsorption performance. This relationship underscores the crucial role of pH in optimizing the adsorption process.

The higher pH_zpc_ than that of CoZr-LDH without any graphene indicates the presence of a significant share of oxygen functional groups.

#### 3.1.4 After adsorption study

**Fig. 12:**
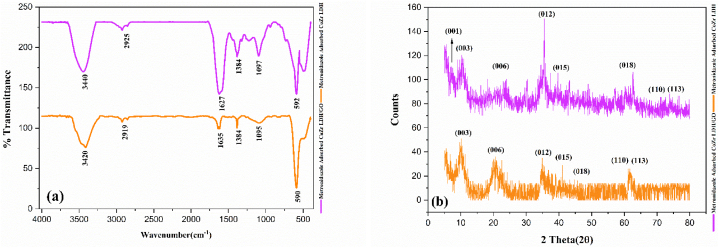
a), IR spectra of Co-Zr LDH (orange spectrum), and the Co-Zr LDH/GO nanocomposite (purple spectrum) after metronidazole adsorption. b) XRD patterns of Co-Zr LDH (orange), and Co-Zr LDH/GO nanocomposite (purple) after adsorption of metronidazole.

After exposing the two adsorbent samples (CoZr LDH and CoZr LDH/GO) in a metronidazole-contaminated environment under optimal conditions (pH 7, concentration of 1500 ppm, and contact time of 30 min), IR and XRD analyses were repeated for both samples, with the results presented in Fig. 12.

Fig. 12 a and 12. b show, respectively, the IR spectrum and XRD pattern for both CoZr LDH and CoZr LDH/GO samples after metronidazole adsorption.

In Fig. 12 a, the orange spectrum corresponds to the CoZr LDH sample, where the peaks at 590 cm⁻^1^ are attributed to metal-oxygen bonds. These peaks show increased intensity compared to Fig. 5. (IR spectrum of CoZr LDH before adsorption), likely due to the formation of metal-oxygen bonds within the structural oxygen of metronidazole. The peaks at 1095 cm⁻^1^ and 1384 cm⁻^1^ correspond to the tensile and flexural vibrations of C-O, while the peak at 1635 cm⁻^1^ is related to C=C vibrations. The bending vibrations of OH are shown at 3420 cm⁻^1^.

In the purple spectrum corresponding to CoZr LDH/GO, the prominent peak at 592 cm⁻^1^ is attributed to metal-oxygen bonds between metronidazole and LDH, which shows a significant increase in intensity compared to Fig. 5. This provides clear evidence of metronidazole adsorption. The peaks at 1097 cm⁻^1^ and 1384 cm⁻^1^ are related to the tensile and flexural vibrations of C-O, while the peak at 1627 cm⁻^1^ corresponds to C=N vibrations. The peak at 2925 cm⁻^1^ is associated with C-H bonds from graphene, and finally, the peak at 3440 cm⁻^1^ represents the flexural vibrations of OH [63].

In the XRD patterns shown in Fig. 12b, the orange pattern exhibits all the peaks identical to those in the CoZr LDH pattern presented in Fig. 6, which were previously discussed. However, the key difference is the reduction in intensity of all the peaks, which could be attributed to the decrease in crystallinity of the phase related to the layers as a result of metronidazole adsorption. Additionally, a slight broadening of the peaks is observed, which may be due to changes in the interlayer spacing of the LDH. This also serves as further evidence of metronidazole adsorption. In Fig. 12b, the purple XRD pattern for the CoZr LDH/GO sample after metronidazole adsorption shows a reduction in the intensity of all peaks, especially the 001 peak at 2θ = 10, which corresponds to GO. This clearly indicates the significant role of GO in metronidazole adsorption, aligning well with the findings and results presented in previous sections. Additionally, a slight shift of the peaks toward higher 2θ values, along with noticeable broadening, suggests changes in the interlayer spacing of the LDH, which is attributed to metronidazole adsorption.

### 3.2 Comparison

Several previous studies have shown appropriate results for MNZ adsorption with different adsorbents as shown in Table 4 and compared to the results of this work.

**Table 4:** Comparison between different adsorbents for metronidazole adsorption.

Adsorbent	q_max_(mg/g)	Ref.
FeNi_3_/SiO_2_/CuS	135.135	[7]
MnAl-LDH	62.804	[18]
MgAl-LDH	62.8	[29]
CoFe_2_O_4_/activated carbon@chitosan	36.897	[17]
UiO-66-NH_2_	217	[15]
Urea-MIL-101	188	[30]
Active carbon	22–152	[27]
CoZr-LDH	189.1376	Our work
CoZr-LDH/GO nanocomposite	906.5688	Our work

### 3.3 Modelling results

#### 3.3.1 Quantum calculations

**Fig. 13:**
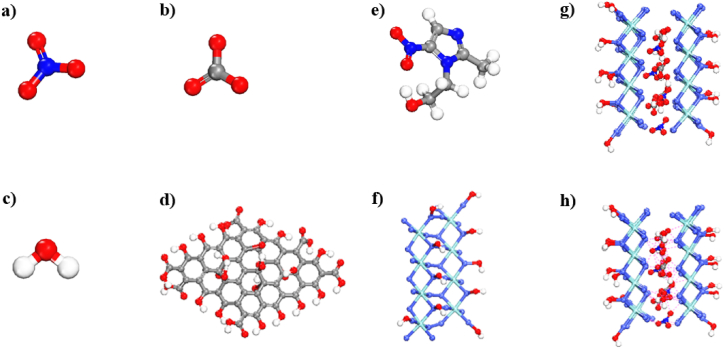
The optimized structures of a); NO_3_^−^, b); CO_3_^2−^, c); H_2_O, d); GO, d); Met, e); Zr-Co, and CoZr LDH (NO_3_^−^, CO_3_^2−^, H_2_O) acquired from DFT-D method using DMol^3^ module in the Materials Studio 2017 software. The gray, red, cyan, blue, and white colored balls represent carbon, oxygen, cobalt, zirconium, nitrogen, and hydrogen atoms respectively [64].

**Fig. 14:**
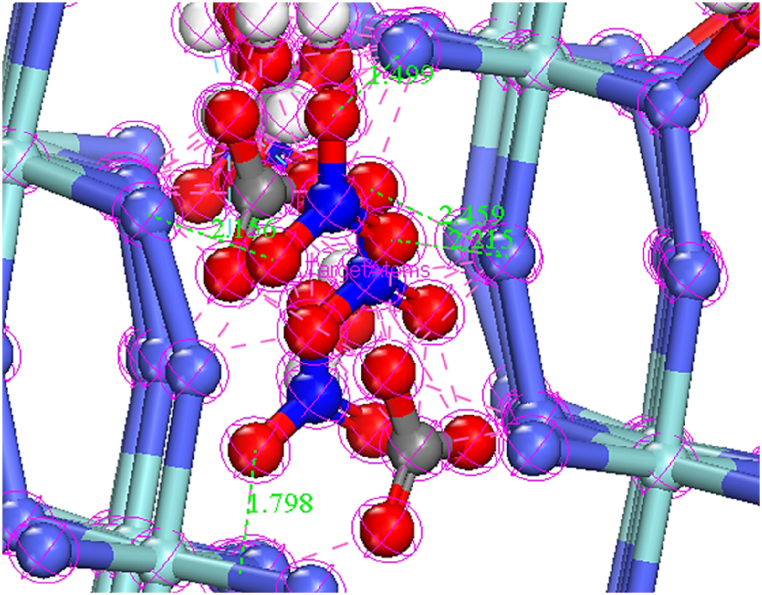
Top view of CoZr-LDH (NO_3_^−^, CO_3_^2−^, H_2_O) with the distances less than 3.00 Å between the N atoms of NO_3_^−^ or CO_3_^2−^ and the Co atom of CoZr-LDH.

Quantum calculations of NO_3_^−^, CO_3_^2−^, H_2_O, GO, metronidazole (MNZ), CoZr, and CoZr-LDH (NO_3_^−^, CO_3_^2−^, H_2_O) were applied to obtain geometric structures by DMol^3^ module based on DFT-D correction [64] and all optimized (relaxed) structures of the compounds by using DFT-D [65] are presented in Fig. 13a–h. The findings showed that the distances between N atoms of NO_3_^−^/or CO_3_^2−^ and the Co atoms of CoZr-LDH were less than 3.00 Å (Fig. 14).

#### 3.3.2 Monte Carlo adsorption locator calculations of GO on CoZr-LDH (CoZr-LDH/GO)

**Fig. 15:**
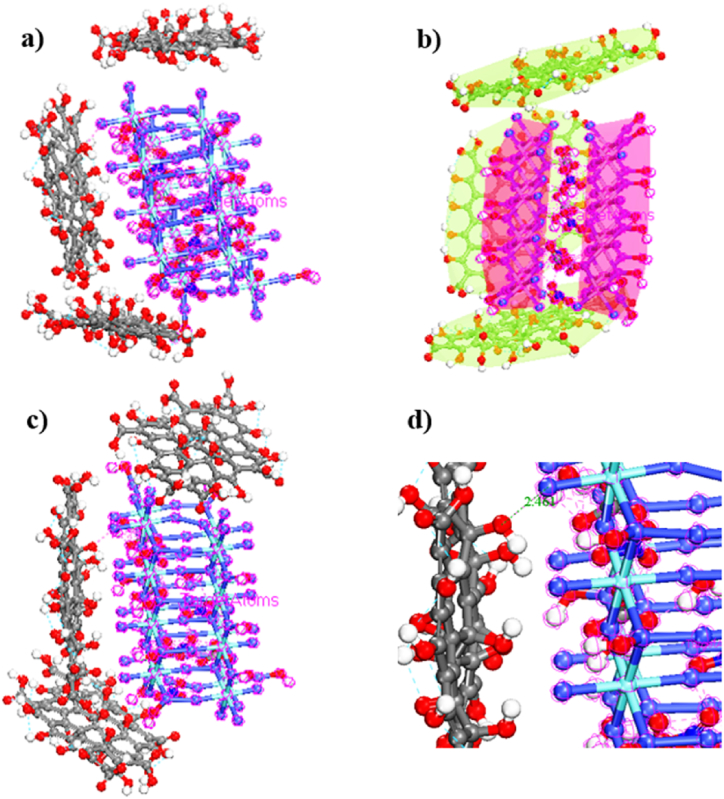
The adsorption of 3 molecules of GO on CoZr-LDH (a–c), side view of the distance between GO and CoZr-LDH.

To form the CoZr-LDH/GO nanocomposite and calculate total energies, the Monte Carlo adsorption locator module was constructed with Forcefield; universal was used with a mixture of 3 GO molecules, and the results are illustrated in Fig. 14a–d. GOs were adsorbed on CoZr-LDH via possible interactions including hydrogen bonds, covalent, electrostatic interactions, and van *der Waals* (vdW) interactions (Fig. 15a–c) [66]*.* The adsorption energy (E_ad_) of GO on CoZr-LDH was −1.633 × 10^5^ kcal/mol. The results indicated that the average distances between GO and CoZr-LDH were less than 3.00 Å when O atoms from –OH and Co atoms were used (Fig. 15d), indicating that oxygen atoms and cobalt play major roles in chelation. The negative data showed that interaction and adsorption of GO on CoZr-LDH were spontaneous behaviors [67,68].

#### 3.3.3 Monte Carlo adsorption locator calculations of MNZ on CoZr-LDH and CoZr-LDH/GO

**Fig. 16:**
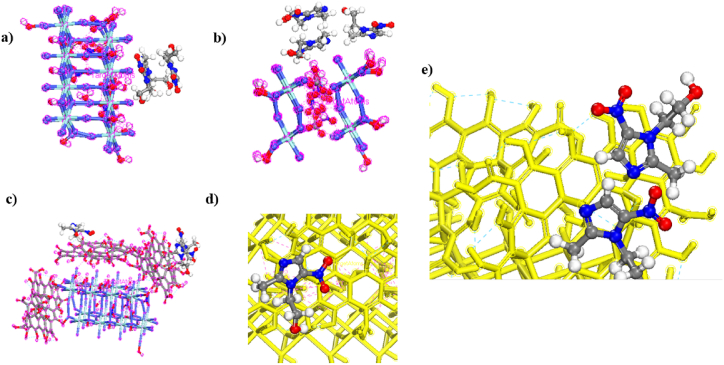
Stable configurations of three molecules of MNZ adsorption on (a–b); CoZr-LDH and (c); configurations of three molecules of MNZ adsorption on CoZr-LDH/GO nanocomposite (d–e); Close-up view of three molecules of MNZ adsorption on CoZr-LDH/GO by a locator module.

The adsorption behavior of MNZ (three molecules) on CoZr-LDH and CoZr-LDH/GO was evaluated by a locator module and a computational force field, which are universal in Materials Studio 2017 software [69,70]. The stable structures are represented in Fig. 16a–e. The results show that in the absence of GO, the configurations obtained with MNZ parallel to the surface of CoZr-LDH were the most stable structures and MNZ was concentrated together on CoZr-LDH (as observed in Fig. 16a–b). The E_ads_ for MNZ adsorption on CoZr-LDH was −250.884 kcal/mol. Moreover, the adsorption energy (ΔE) of MNZ on CoZr-LDH/GO was greater than on CoZr-LDH due to the π-π stacking, hydrogen bonding, and electrostatic interactions between MNZ and the GO sheets (as observed in Fig. 15c–e) [36]. The adsorption energy (*E*ad) of MNZ on CoZr-LDH/GO was approximately −270.662 kcal/mol. The theoretical results were in agreement with the experimental data [71].

## 4 Conclusion

In this study, we successfully synthesized CoZr-LDH and CoZr-LDH/GO nanocomposite adsorbents for the first time using a green, solventless mechanochemical approach and an autoclave method, respectively. These adsorbents were applied for the adsorption of metronidazole (MNZ) from aqueous solutions. The findings reveal that compositing CoZr-LDH with graphene oxide significantly enhanced the BET surface area, increasing from approximately 32.995 m^2^/g for CoZr-LDH to 297.47 m^2^/g for the CoZr-LDH/GO nanocomposite. The adsorption capacities also exhibited a substantial improvement, with CoZr-LDH adsorbing 189.1376 mg g^−1^ and CoZr-LDH/GO achieving 906.5688 mg g^−1^. The superior adsorption performance of CoZr-LDH/GO was attributed to the higher number of oxygen functional groups provided by the graphene oxide and π-π interactions between the metronidazole ring and the hexagonal graphene lattice. Additionally, some MNZ molecules were retained through space-filling mechanisms due to the extensive surface network of the GO.

The kinetic analysis showed that the pseudo-second-order model better described the adsorption behavior compared to the pseudo-first-order model. Among the three isotherm models evaluated, the Redlich-Peterson model provided the most accurate fit, supported by the highest R^2^ values (0.9602 for CoZr-LDH and 0.997 for CoZr-LDH/GO) and the lowest χ^2^ values (1.537 for CoZr-LDH and 0.1362 for CoZr-LDH/GO). The non-ideal monolayer adsorption observed, with β values of 0.75 for CoZr-LDH and 0.66 for CoZr-LDH/GO, indicated that multilayer adsorption is the predominant mechanism for MNZ removal by these nanocomposites.

Quantum calculations, using the DMol³ module with DFT-D corrections, optimized the structural geometries, while Monte Carlo adsorption locator simulations, utilizing a universal force field in Materials Studio 2017, identified favorable adsorption sites and energies. Results from these simulations indicated that MNZ adsorbed more strongly on the CoZr-LDH/GO nanocomposite than on CoZr-LDH, emphasizing the enhanced performance due to the presence of GO.

Innovations and Comparison: This research presents several novel contributions, including the first-time synthesis of CoZr-LDH/GO via a solventless, green method and the detailed computational modeling of adsorption mechanisms. The comparative analysis using Monte Carlo methods to predict adsorption behavior is also pioneering and underscores the greater adsorption potential of CoZr-LDH/GO compared to similar studies reported in the literature.

Limitations and Future Work: While the study provides robust insights into the adsorption mechanisms of MNZ on CoZr-LDH and CoZr-LDH/GO, limitations include the controlled laboratory conditions under which experiments were conducted. Future work should focus on evaluating the adsorption performance of these materials in real contaminated aquatic environments to validate their practical applications and enhance their relevance for environmental remediation.

## CRediT authorship contribution statement

**Edris Jamshidi:** Writing – original draft, Visualization, Supervision, Project administration, Methodology, Formal analysis, Data curation. **Fateme Fathabadi:** Software, Formal analysis, Data curation. **Faranak Manteghi:** Writing – review & editing, Supervision, Project administration, Conceptualization. **Rahime Eshaghi Malekshah:** Writing – original draft, Software, Methodology.

## Declaration of competing interest

The authors declare that they have no known competing financial interests or personal relationships that could have appeared to influence the work reported in this paper.
